# Pregnancy after living kidney donation, a systematic review of the available evidence, and a review of the current guidance

**DOI:** 10.1111/ajt.17122

**Published:** 2022-08-03

**Authors:** Maria Pippias, Laura Skinner, Marlies Noordzij, Anna Varberg Reisæter, Daniel Abramowicz, Vianda S. Stel, Kitty J. Jager

**Affiliations:** ^1^ Bristol Medical School: Population Health Sciences University of Bristol Bristol UK; ^2^ North Bristol NHS Trust, Renal Unit Bristol UK; ^3^ Bristol Medical School: Translational Health Sciences University of Bristol Bristol UK; ^4^ Department of Internal Medicine University Medical Center Groningen Groningen The Netherlands; ^5^ Department of Transplantation Medicine Oslo University Hospital Rikshospitalet Norway; ^6^ Department of Nephrology Antwerp University Hospital Edegem Belgium; ^7^ ERA Registry, Department of Medical Informatics Amsterdam Public Health Research Institute, Amsterdam UMC‐Location AMC, University of Amsterdam Amsterdam The Netherlands

**Keywords:** donor nephrectomy, donor outcomes, kidney transplantation, living donor, pre‐eclampsia

## Abstract

Understanding and communicating the risk of pregnancy complications post‐living kidney donation is imperative as the majority of living kidney donors (LKD) are women of childbearing age. We aimed to identify all original research articles examining complications in post‐donation pregnancies and compared the quality and consistency of related guidelines. We searched Embase, MEDLINE, PubMed, society webpages, and guideline registries for English‐language publications published up until December 18, 2020. Ninety‐three articles were screened from which 16 studies were identified, with a total of 1399 post‐donation pregnancies. The outcome of interest, post‐donation pregnancy complications, was not calculable, and only a narrative synthesis of the evidence was possible. The absolute risk of pre‐eclampsia increased from ~1%–3% pre‐donation (lower than the general population) to ~4%–10% post‐donation (comparable to the general population). The risks of adverse fetal and neonatal outcomes were no different between post‐donation and pre‐donation pregnancies. Guidelines and consensus statements were consistent in stating the need to inform LKDs of their post‐donation pregnancy risk, however, the depth and scope of this guidance were variable. While the absolute risk of pregnancy complications remains low post‐donation, a concerted effort is required to better identify and individualize risk in these women, such that consent to donation is truly informed.

## Abstract 2

This systematic review of complications of pregnancies after living kidney donation shows low absolute risk of complications and variability in the depth and scope of guidance provided but consistency in stating the need to inform donors of their post‐donation pregnancy risks.

**Abbreviations**
BMI: body mass index
CI: confidence interval
CKD: chronic kidney disease
EMG: ethnic minority groups
ESKD: end‐stage kidney disease
GFR: glomerular filtration rate
LKD: living kidney donors
MeSH: medical subject headings
OR: odds ratio

## 1 INTRODUCTION

Living kidney donor (LKD) transplants are a valuable resource. They have a greater survival advantage compared to deceased donor transplantation or remaining on dialysis,^1^ they are cost‐efficient,^2^ and help combat the deficit in available organs. It was long believed that kidney donation did not infer a risk to the health of the LKD. Recently, some studies have called this into question,^3^, ^4^ though this is not a universal finding^5^, ^6^ and the absolute risk to LKDs remains low.

Kidney donation results in a degree of kidney function loss; by 10 years post‐donation 12% of LKDs will have an estimated glomerular filtration rate (GFR) of <60 ml/min.^7^ Piccoli et al., demonstrated that even early stages of chronic kidney disease (CKD) can lead to an increased risk of adverse events in pregnancy.^8^ As the majority of LKD are women of childbearing age,^9^ it is vital that a clear picture of the risks associated with pregnancy post‐kidney donation is obtained and that the available guidance is comprehensive.

We performed a systematic review to answer the question “*Are LKD at an increased risk of pregnancy‐induced complications following a donor nephrectomy, compared to the risks of pregnancy‐induced complications in healthy women who have not undergone a donor nephrectom*y*?*” Second, we identify guidelines, consensus statements, and expert opinions which include the issue of pregnancy in LKD. Third, we identify areas which could be addressed in guidelines focusing on pregnancy in LKD.

## 2 METHODS

### 2.1 Systematic review of pregnancy outcomes in LKD

On December 18, 2020, a PubMed, EMBASE, and Cochrane library search was performed for the medical subject headings (MeSH) terms and text words for kidney transplantation, living kidney donor combined with the MeSH terms for pregnancy; maternal, fetal, pregnan*, nephrect*, kidney, rena*, nephrol*, postdona*, and donation (Figure S1).

All study designs which included LKD with a post‐donation pregnancy with any maternal or fetal complication were included. Publications were limited to human‐based, English‐language studies, without time limits for the length of follow‐up or publication date. Nephrectomies for non‐donation causes were excluded. Initial screening of study titles and abstracts was independently performed by three authors (MP, LS, and KJJ) using Rayyan software.^10^ Disagreements were resolved by consensus or discussion with a third reviewer. Eligibility assessment of the full article and data extraction was performed independently by two authors (MP and MN, LS, or KJJ). A pre‐specified list of maternal, fetal, and neonatal outcomes was extracted as proportions and/or odds ratios without limitations on outcome domains, that is, all time points were included (Table S1, Appendix S1). The reference lists of relevant publications were hand‐searched. Study authors were directly contacted when clarification was required, that is, uncertainty over study duplication^11^ or missing data.^12^

MP and LS independently assessed the risk of bias pertaining to a study's participant selection, ascertainment of exposure, and assessment of outcomes using the Risk Of Bias In Non‐randomized Studies of Interventions (ROBINS‐I) tool.^13^ Consensus was reached using dialogue. The plots for visualizing the risk‐of‐bias assessments were created using the robvis web app.^14^ Certainty in the study was assessed using the GRADE approach to rating certainty.^15^

Populations were grouped as pre‐donation, post‐donation, and general population. Bar charts were used to graphically represent the percentage of hypertensive disorders in pregnancy and fetal complications by population group as reported in studies deemed moderate/low risk of bias. A lack of consistent outcome definitions and the heterogeneity of the controls inhibited a pre‐specified meta‐analysis. Heterogeneity was informally investigated by structuring figures and tables by study design.^16^ See the PRISMA 2020 checklist (Appendix S2).

### 2.2 Guidelines, consensus statements, and expert opinions

A PubMed search was performed for the MeSH terms and text words for guidelines (practice guideline(s), clinical practice guideline(s), standards, consensus statement, and consensus) combined with the MeSH terms for kidney transplantation and living kidney donor. Furthermore, national renal bodies' websites were examined along with a Google search and a hand‐search of the relevant guidelines' reference lists. Based on the available studies of pregnancy outcomes in LKD, which from 2009 were far superior in terms of study design to prior studies, we limited the inclusion of guidelines to those published from 2010 onwards. Where two guidelines from the same group had been published since 2010, we included the most recent.

## 3 RESULTS

### 3.1 Systematic review of pregnancy outcomes in LKD

**FIGURE 1:**
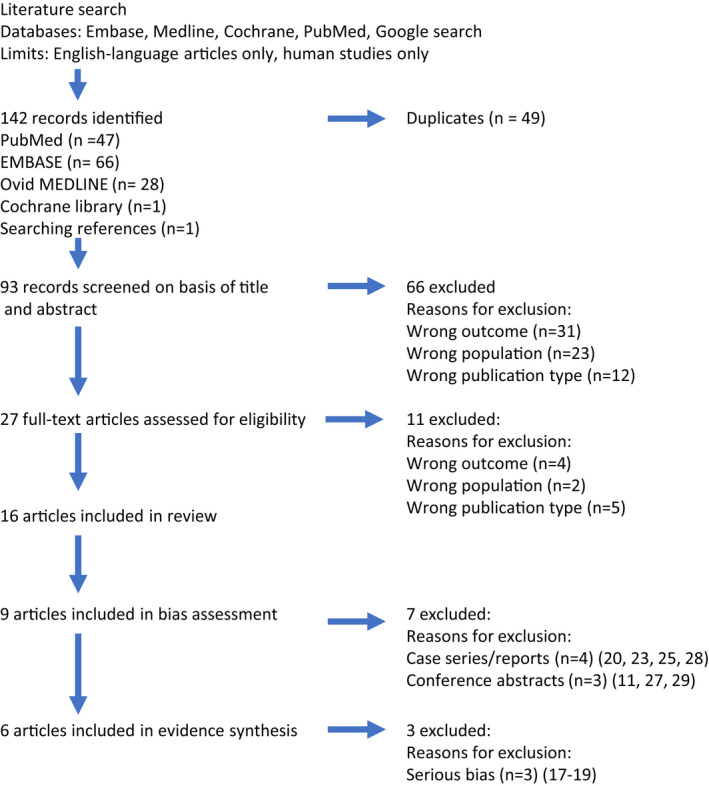
Selection of studies and reasons for exclusion at each stage of the systematic review.

**FIGURE 2:**
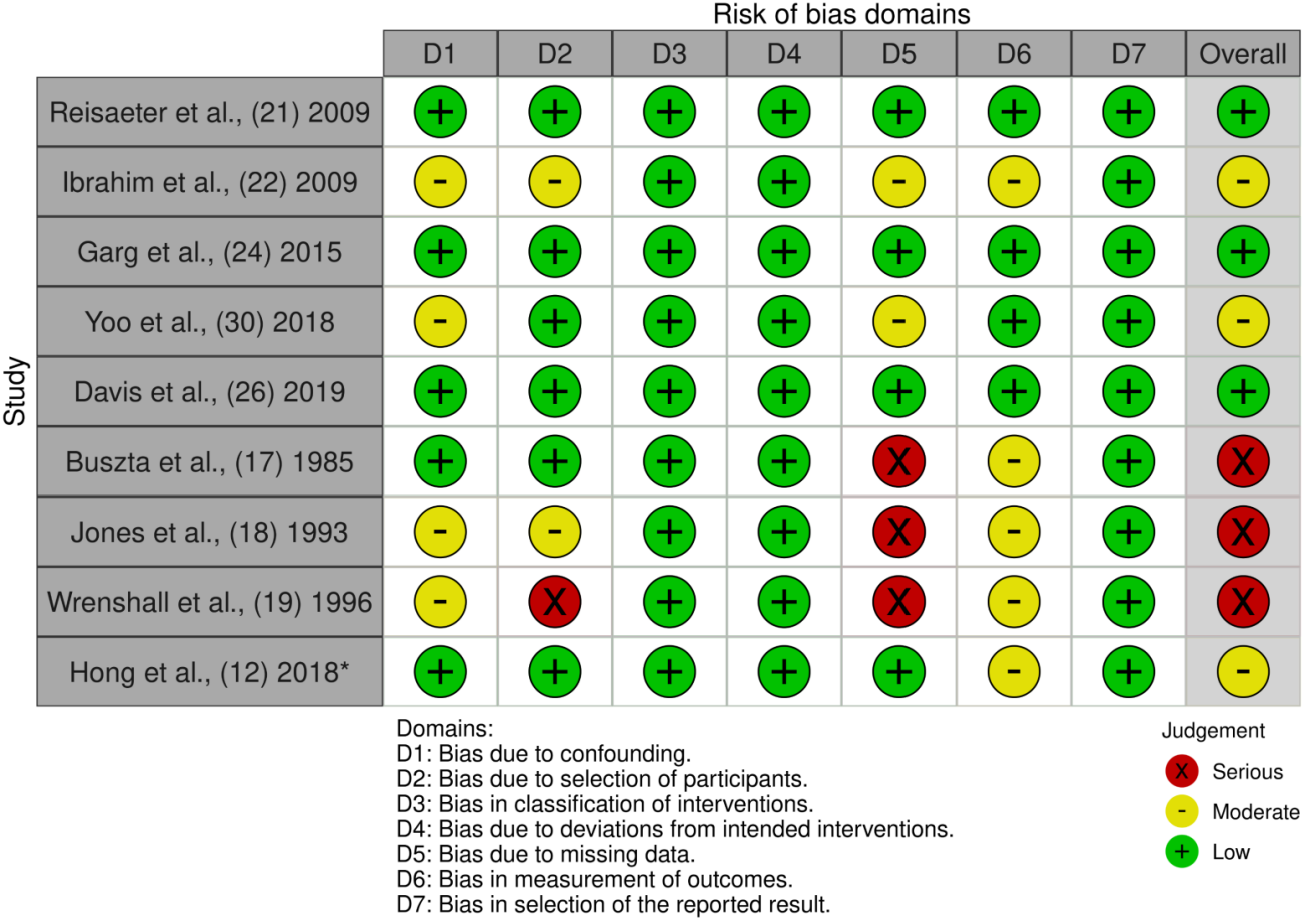
Traffic light plot of the domain level, risk of bias judgments for each individual retrospective cohort, and matched‐controlled studies and retrospective descriptive studies without a control group. *Additional information on missing data obtained by direct contact with author.

**FIGURE 3:**
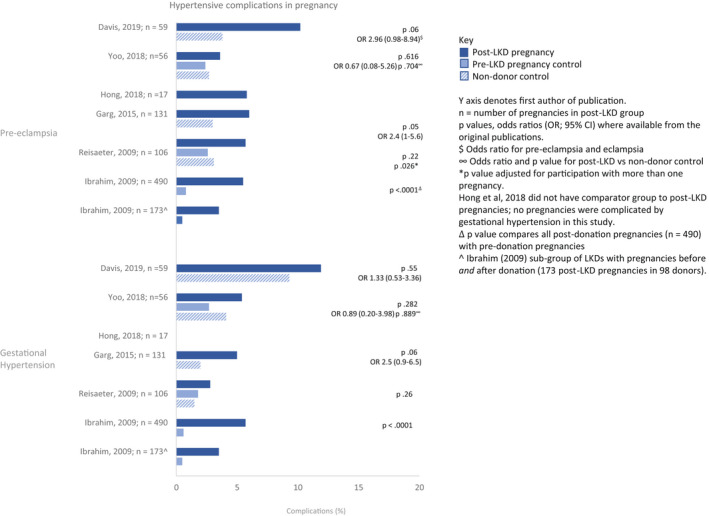
Hypertensive complications in pregnancy in the pre‐donation, post‐donation, and general population presented by study for pre‐eclampsia (upper panel) and gestational hypertension (lower panel).

**FIGURE 4:**
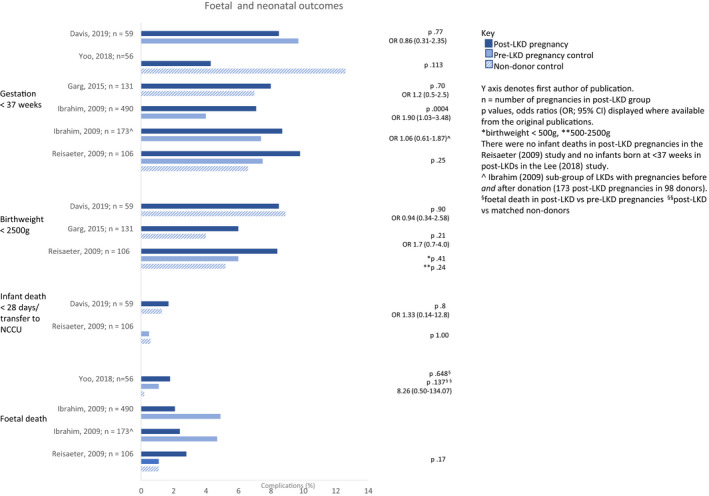
Fetal complications in pregnancy in the pre‐donation, post‐donation, and general population presented by study. From top to bottom: gestation <37 weeks, birthweight <2500 g, infant death <28 days/transfer to NCCU, and fetal death.

Of the 93 articles identified, 66 were excluded based on the initial title/abstract screening, leaving 27 for full screening; 16 articles were included in the systematic review (Figure 1).^11^, ^12^, ^17^, ^18^, ^19^, ^20^, ^21^, ^22^, ^23^, ^24^, ^25^, ^26^, ^27^, ^28^, ^29^, ^30^ These were published over 35 years (1985–2020), from eight countries (Table 1). Four case studies/series were identified but not discussed further.^20^, ^23^, ^25^, ^28^ Risk‐of‐bias, varied from low^21^, ^24^, ^26^ to moderate^12^, ^22^, ^30^ to serious^17^, ^18^, ^19^ (Figure 2). Studies with a low risk of bias were broadly consistent in the direction of their findings. The certainty in the evidence was deemed low for the outcomes gestational hypertension and pre‐eclampsia (Table 2) and very low for gestation (<37 weeks) and birthweight (<2500 g, Table 2). The percentage of hypertensive disorders in pregnancy and fetal complications in the pre‐donation, post‐donation, and general population are summarized in Figures 3 and 4, respectively. Although all pregnancy complications were included, the predominant complications identified were hypertensive disorders of pregnancy, proteinuria, and gestational diabetes.

**TABLE 1:** Overview of the 12 studies identified, excluding case reports/series, which reported on the pregnancy outcomes in living kidney donors (LKD)

Number of LKD	Number of post‐donation pregnancies	Number of control pregnancies	Donation period	Time from donation to pregnancy	Setting	Study design	Results	Conclusion	Limitations/comments
Retrospective cohort and matched cohort studies									
1. Reisaeter et al.,2009^21^									
326	106 pregnancies in 69 LKD	620 in LKD pre‐donation & 21 000 in non‐LKD	1967–2002	5 years (3.4) (mean, SD)	University Hospital, Norway	Medical Birth Registry. Controls: pre‐donation pregnancies and random sample of women from the Medical Birth Registry. Before‐and‐after‐design.	The occurrence of pre‐eclampsia was more common after donation than before (in adjusted analysis only). Pre‐donation: 2.6%, post‐donation: 5.7%, general population: 3.1%. Complications pre‐ versus post‐donation: chronic HTN: 0.2 versus 0.9%; gestational HTN: 1.8 versus 2.8%; pre‐eclampsia: 2.6 vs. 5.7%; birth weight < 500 g: 0.5 versus 0.9%; gestational age < 22 weeks: 0.3 versus 1.0%; stillbirth: 1.1 versus 2.8%; neonatal death (<28 days since birth): 0.5 versus 0.0%.	*“No differences observed in the occurrence of adverse pregnancy outcomes in donors. The finding of more frequent preeclampsia post pregnancy should be interpreted with caution given the low numbers. Supports idea that pregnancy after donation is safe, women of fertile age should not be discouraged, careful monitoring is warranted. Potential donors should be informed of the increased risk of preeclampsia with reference to the possible effect on the infant”*	98% participants reported as white. Outcomes defined. % missing data not reported.
2. Ibrahim et al. 2009^22^									
1589	3213 pregnancies in 1085 LKD	204 pre‐donation pregnancies in 98 LKD who also had 173 post‐donation pregnancies and 2519 pre‐donation pregnancies in 846 LKD	1963–2007	Not stated	Nephrology Clinic, Minnesota, US	Retrospective postal questionnaire with telephone follow‐up of non‐responders. Controls: Pre‐donation pregnancies. Before‐and‐after‐design.	Post‐donation pregnancies had worse outcomes compared to pre‐donation pregnancies. Complications pre‐ versus post‐donation: gestational HTN: 0.5 versus 3.5%; pre‐eclampsia: 0.5 vs. 3.5%; gestational diabetes: 0.5 versus 0.6%; proteinuria: 1.5 versus 4.6% premature delivery: 7.4 versus 8.7% (OR: 1.06; 95% CI: 0.61; 1.87) fetal loss: 21.1 versus 24.3%; (OR: 1.35; 95% CI: 0.58; 3.18).	Post‐donation pregnancy is associated with a higher likelihood of adverse outcomes whether a LKD had a pre‐donation pregnancy or not. The frequency of adverse outcomes is similar and in some Domains better than those reported in the general population. Importantly, there is a 7‐fold increase in the adjusted risk of preeclampsia in post‐donation pregnancies.	75% response rate. Self‐reported outcomes. Subject to selection bias and recall bias, survival bias, response bias. 24–39% loss to follow‐up rate. 97% of responders reported as white. Outcomes defined.
3. Garg et al.,2015^24^									
85	131 pregnancies in 85 LKD	788 pregnancies in 510 non‐donors	1992–2009		Ontario, Canada	Retrospective cohort study of LKD & matched healthy controls identified via provincial health care databases	Post‐donation LKD pregnancies had increased frequency of gestational hypertension and preeclampsia compared to non‐donor pregnancies. Complications post‐donation non‐donors versus post‐donation LKD: Gestational HTN: 2 versus. 5%, (OR: 2.5, 95% CI: 0.9; 6.5); Pre‐eclampsia: 3 versus 6% (OR: 2.4, 95% CI: 1.0; 5.6); Birth weight <2.5 kg: 4 versus 6%, (OR: 1.7, 95% CI: 0.7; 4.0) Gestational age < 37 weeks: 7% versus 8%, (OR: 1.2, 95% CI: 0.5; 2.5).	Gestational HTN and preeclampsia were more likely to be diagnosed in LKD than in matched non‐LKD with similar indicators of health.	Women within the general population with an episode of gestational diabetes, gestational hypertension or pre‐eclampsia were excluded. 70% participants reported as white. Outcomes defined.
4. Yoo et al., 2018^30^									
225	56 pregnancies in 39 LKD	437 pregnancies in non‐donor control group	198 572–2014	Not stated	Korea	Matched case controls: before and after donation and matched non‐donor pregnancies.	Gestational hypertension (5.4% vs. 2.7%, *p* = .282) and preeclampsia (3.6% vs. 2.4%, *p* = .616) rates were not significantly different in post donation pregnancies compared to pre‐donation pregnancies. Cesarean section rate was higher post‐donation (33.9% vs. 21.6%, *p* = .005). Donors were older with higher BMIs in post‐donation pregnancies. Fetal death was similar between post‐ and pre‐donation pregnancies (1.8% vs. 1.1%, *p* = .648).	Adverse maternal outcomes in pregnant women after kidney donation were not different from those before kidney donation.	Background states “outcomes in Asian population not well established”, therefore implying this study has a predominantly Asian population though ethnicity is not explicitly reported. 54% LKD response rate to postal questionnaire.
5. Davis et al., 2019^26^									
59	59 first pregnancies in 59 LKD	236 pregnancies in non‐donor control	Not stated (deliveries between 1996 and 2015)	Mean ± SD 2.7 ± 1.8 years	Utah & Idaho, USA	Matched cohort study of first pregnancy in LKD & matched healthy controls identified via Intermountain Healthcare Enterprise Data Warehouse.	Post‐donation LKD pregnancies had an increased odds ratio of pre‐eclampsia/eclampsia compared to non‐donor pregnancies. Complications healthy controls versus post‐donation LKD: Gestational HTN: 9.3 versus 11.9%, (OR: 1.33, 95% CI: 0.53; 3.36) Pre‐eclampsia/eclampsia: 3.8 versus 10.2% (OR: 2.96, 95% CI: 0.98; 8.94); Gestational diabetes: 6.4 versus 5.1% (OR: 0.80, 95% CI 0.23; 2.79); Birth weight <2.5 kg: 8.9 versus 8.5%, (OR: 0.94, 95% CI: 0.34; 2.58) Transfer to acute facility or death <28 days from birth: 1.3 versus 1.7%, (OR: 1.33, 95% CI: 0.14; 2.6);	“ *…the risk of pregnancy‐associated complications following kidney* *donation is small. While there appears to be an increased risk of preeclampsia, there is no* *increased risk of preterm delivery, delivery* via *cesarean section or low birth weight. As such, the majority of female donors can expect a normal pregnancy. Potential donor candidates should be counseled regarding the possible complications of pregnancy including the risk of preeclampsia and gestational hypertension, especially in younger nulliparous women.”*	Identification of donation by ICD‐9 code; Data on smoking and kidney function unavailable; Risk of insufficient power; Limited information on baseline characteristics: the donor group may have been (much) healthier leading to confounding by indication. No information on missing data available. >90% participants reported as white. Outcomes defined
Retrospective descriptive studies without control group									
6. Buszta et al., 1985^17^									
23	39 pregnancies in 23 LKD	0	Not stated	2 weeks to 9 years (range)	University Hospital, US	Retrospective review of prenatal and delivery records.	Transient proteinuria seen in 9 women. No other complications seen. However, it was not clear from the methods if pre‐eclampsia and gestational hypertension were included as potential outcomes.	*“We think that the risk of donor nephrectomy is small and that the question of progressive renal injury due to hyperfiltration remains unanswered. We are encouraged that there does not seem to be an increased risk of pregnancy in women after donor nephrectomy.”*	One pregnancy was counted twice as the woman delivered twins. One woman was not included in the miscarriage outcomes as she was pregnant at the time of completing the survey. Outcomes not defined. Ethnicity not reported.
7. Jones et al., 1993^18^									
14	25 pregnancies	0	Not stated	Not stated	Nephrology Clinic, Minnesota, US	Retrospective telephone survey.	Two miscarriages (8%). No other adverse events.		Subject to selection bias and recall bias. No data on kidney function.
8. Wrenshall et al., 1996^19^									
144	45 pregnancies in 33 LKD	0	1985–1992	Not stated	Nephrology Clinic, Minnesota, US	Retrospective postal questionnaire (self‐reported outcomes).	75% event‐free pregnancies carried to term. Complications post ‐donation: Miscarriage, 13.3%; Pre‐eclampsia, 4.4% (2/45); Gestational HTN, 4.4%; Proteinuria, 4.4%; Tubal pregnancy, 2.2%; Overall morbidity, 8.8%.	*“Donor nephrectomy is not detrimental to the prenatal course or outcome of future pregnancies”*	65.5% response rate. Subject to selection bias and recall bias. Some overlap with the LKD included in the study by Jones et al. No data on kidney function. Ethnicity not reported. Outcomes not defined.
9. Hong et al., 2018^12^									
56 female LKD	17 pregnanciesin 11 women	0	1990–2015	Not stated	Single center, Hong Kong	Retrospective cohort of 83 LKDs, of which 56 were women. No control group.	Of the 11 pregnant LKD post donation with 17 pregnancies: 0% hydronephrosis; 1% urinary tract infection; 11% pre‐eclampsia; 10% gestational diabetes; 0% gestational hypertension; 22% fetal loss	*“The small sample size* *undermines the ability to infer the actual (incidence of complications)”*	Retrospective cohort with low number of post‐donation pregnancies with incomplete data on pregnancy outcomes within this sub‐set of the cohort.* 97.6% Chinese ethnicity. Outcomes not defined. 73/83 LKDs had donor follow‐up. Unclear how many may be women of childbearing age. Outcomes on 1 pregnancy missing.
Conference abstracts									
10. Ibrahim et al., 2017^11^									
2360	311	0	1963–2013	Not stated. Follow‐up 25 ± 12 years from donation, 21 ± 12 years from pregnancy.	Nephrology Clinic, Minnesota, US	Retrospective questionnaire	Outcomes in LKDs with pre‐eclampsia (*N* = 38) versus LKD without pre‐eclampsia (*N* = 273): LKDs with pre‐eclampsia were younger at time of donation (23 ± 4 vs. 27 ± 6, *p* < .001) and a lower proportion had a pregnancy prior to donation (24% vs 54%, *p* < .001). ESKD: 1 versus 0; Hypertension: 55% versus 27%; Diabetes mellitus: 18% versus 7%; Proteinuria: 18% versus 6%. LKDs with pre‐eclampsia were more likely to develop hypertension and proteinuria.	*“LKDs with preeclampsia after donation are more likely to become proteinuric, hypertensive and their eGFR may decline at a faster rate. These outcomes are, however, comparable to what is seen in women with a history of PE and two kidneys and also male donors.”*	Abstract. Note this includes the 173 post‐donation pregnancies previously reported in.^22^ Ethnicity not reported.
11. Lee et al., 2019^27^									
248	248	5 665 539	2005–2014	Not stated	Multi‐center, California, Florida, and New York, US	Retrospective cohort. Controls: all other pregnancies 2005–14 listed on the Healthcare Cost and Utilization Project State Inpatient Databases.	No difference in risk for hypertensive disorders of pregnancy. Donors had increased risk of membrane‐related disorders (OR 1.48, 1.01–2.11, *p* = .045) and a decreased risk of gestational diabetes (OR 0.53, 0.30–0.96, *p* = .037).	*“Future research should assess risk against matched controls to elucidate the impact of kidney donation on women of child‐bearing age.”*	Abstract. 10.9% of participants reported as Black.
12. Kara‐Hadj Safi et al., 2019^29^									
66	5 pregnancies	0	2007–2017	Not stated	Single‐center, Tlemcen, Algeria	Descriptive study of 100 (male and female) LKDs at least 1‐year post‐donation.	No maternal or fetal complications seen.		Abstract. Unable to contact corresponding author to clarify numbers.

**TABLE 2:** Outcomes and certainty of evidence

Maternal outcomes	Effect	Number of pregnancies/events overall/events in donors	Number of studies	Certainty in the evidence
Pre‐eclampsia	The occurrence of pre‐eclampsia was more common in LKD after donation, compared to before donation or in the general population.	27 904/812/56	5	Low ⊕⊕◯◯◯
Gestational hypertension	The occurrence of gestational hypertension was more common in LKD after donation, compared to before donation or in the general population.	27 904/464/55	5	Low ⊕⊕◯◯

Fetal outcomes	Effect	Number of pregnancies/events overall/events in donor pregnancies	Number of studies	Certainty in the evidence
Gestation <37 weeks	The occurrence of delivery <37/40 weeks gestation appears to be more common in LKD after donation, compared to before donation or in the general population.	27 904/1728/90	5	Very low ⊕◯◯◯ due to imprecision and inconsistency
Birthweight <2500 g	The occurrence of a birthweight <2500 g appears to be more common in LKD after donation, compared to before donation or in the general population.	27 904/1221/22	3	Very low ⊕◯◯◯ due to imprecision and inconsistency

*Note*: Certainty in evidence assessed by methodological limitations of the studies, indirectness, imprecision, inconsistency, and likelihood of publication bias.

### 3.2 Maternal outcomes

The initial three studies by Buszta et al.,^17^ Jones et al.,^18^ and Wrenshall et al.,^19^ published over 25 years ago, were all US‐based single‐center retrospective studies, with a study design that was either a review of the medical notes, a telephone‐ or postal‐survey, respectively. The LKD pregnancy outcomes in these studies were compared to general population outcomes and not to well‐matched control groups. In 39 post‐donation pregnancies in 23 LKD studied by Buszta, all women were normotensive during pregnancy.^17^ Similarly, none of the 25 pregnancies in 14 post‐donation women in the Jones cohort were complicated by gestational hypertension.^18^ Wrenshall reported pre‐eclampsia in 4.4% of post‐donation pregnancies as compared to the general population incidence of 6%–8%.^19^ Based on the poor quality of these studies (i.e., high risk of recall‐, survival‐ and response‐bias, small sample sizes, and incomplete data reporting), they were excluded from Figures 3 and 4.

We identified seven retrospective cohort studies with control groups.^11^, ^21^, ^22^, ^24^, ^26^, ^27^, ^30^ Of these, three had a before‐after‐design,^21^, ^22^, ^30^ whereby pregnancy outcomes were assessed in women both before and after LKD and three studies compared the pregnancy outcomes of LKD with ("healthy") controls.^21^, ^22^, ^30^ Reisæter et al., via the Norwegian Renal Registry identified 408 LKD of childbearing age (≤45 years old) between 1967 and 2002.^21^ Using the Norwegian Medical Birth Registry, they identified 326 LKD with pregnancies before and/or after kidney donation. They compared 106 post‐donation pregnancies in 69 LKD to 620 pre‐donation pregnancies, and a random sample of 21 511 general population pregnancies. The incidence of gestational hypertension was similar in all groups (2.8% post‐donation, 1.8% pre‐donation, and 1.5% in the general population, *p* = .26). In the adjusted analysis, pre‐eclampsia occurred at double the rate in post‐donation (5.7% vs. 2.6%, *p* = .026) pregnancies. The mean post‐donation pregnancy age was 31.9 years, versus 25 years in the pre‐donation pregnancy group. The higher rate of pre‐eclampsia remained even after adjustments for maternal age, pregnancy number, and birth year.

Ibrahim et al., presented a before‐and‐after study by means of a postal survey to all 2102 LKD who donated between 1963 and 2007 in Minnesota, US; 75% responded. When comparing post‐donation to pre‐donation outcomes, there was a higher percentage of gestational diabetes (2.7% vs. 0.7%, *p* = .0001), gestational hypertension (5.7% vs. 0.6%, *p* < .0001), pre‐eclampsia (5.5% vs. 0.8%, *p* < .0001), and proteinuria (4.3% vs. 1.1%, *p* < .0001). Overall, post‐donation outcomes were worse than pre‐donation, but similar to general population outcomes. Expanding on the same cohort, the group presented the long‐term consequences of post‐donation pre‐eclampsia in the LKDs (followed up for 21 ± 12 years from the index pregnancy) in a 2017 abstract.^11^ They reported an increased likelihood of proteinuria, hypertension, and eGFR decline in post‐donation pregnancies complicated by pre‐eclampsia, though these risks were comparable to the risks experienced by the general population with pre‐eclampsia.^11^

In a Korean study of 225 LKD, Yoo et al. (2018) compared 56 post‐donation pregnancies in 39 LKD with 370 pre‐donation pregnancies in 186 pre‐LKDs and with 437 propensity‐matched non‐donor pregnancies. There were no significant differences between the incidence of gestational hypertension (5.4% post‐donation vs. 2.7% pre‐donation, *p* = .282; and 4.1% in non‐donors, *p* = .889) or pre‐eclampsia (3.6% post‐donation vs. 2.4% pre‐donation, *p* = .616; and 2.7% in non‐donors, *p* = .704). Post‐donation women were older with a higher body mass index (BMI) and were more likely to undergo cesarean section.^30^

Three studies compared outcomes to the general population. Garg et al. (2015)^24^ compared 131 post‐donation pregnancy outcomes in 85 LKD who donated during 1992–2010 in Ontario, Canada to a healthy general population cohort of 510 women with 788 pregnancies. The risk of gestational hypertension or pre‐eclampsia was higher in LKDs (11% vs. 5%, odds ratio [OR]:2.4, 95% confidence interval [CI]:1.2–5.0, *p* = .01). Older LKD mothers (>32 years) had a significantly higher likelihood of these outcomes (OR:9.4, 95% CI:3.2–27.5). In pregnancies occurring >2 years from donation/cohort entry the odds of gestational hypertension or pre‐eclampsia was 3.6 (95% CI:1.6–8.3) times higher in LKD.

Lee et al. (2019, conference abstract) retrospectively reviewed 5 665 787 pregnancies, from US centers, recorded in the Healthcare Cost and Utilization Project State Inpatient Databases.^27^ The same proportion of hypertensive complications were noted in the 248 LKD post‐donation pregnancies compared to the 5 665 539 non‐donor pregnancies. LKD had an increased risk of premature rupture of membranes and oligohydramnios (OR: 1.48, 95% CI: 1.01–2.11, *p* = .045) but a decreased risk of gestational diabetes (OR: 0.53, 95% CI: 0.30–0.96, *p* = .037).

Davis et al. (2019) using the Intermountain Healthcare Enterprise Data Warehouse, compared pregnancy outcomes in 59 LKD (≥2 years after donation) with 236 age‐ and race‐matched controls.^26^ The risk of pre‐eclampsia/eclampsia was not statistically different between the groups (OR: 2.96, 95% CI: 0.98–8.94, *p* = .06). LKD ≤30 years, had a four‐fold increased risk of pre‐eclampsia/eclampsia (OR: 4.09, 95% CI: 1.07–15.59, *p* = .04). Although the number of women in this sub‐group was not stated and the observed differences may be confounded by the absence of co‐morbidity‐matched controls.

### 3.3 Fetal and neonatal outcomes

In most studies, there were no differences in adverse fetal and neonatal outcomes between post‐donation, pre‐donation, and non‐donor pregnancies (Figure 4). Reisaeter and Garg found similar rates of low birthweight (<2500 g) or premature (delivery <37 weeks) neonates.^21^, ^24^ In the study by Yoo, no neonates from post‐donation pregnancies were of a low birthweight, although it should be mentioned that this was defined as <2800 g, as compared to the more widely adopted threshold of <2500 g.^30^

There were no fetal or neonatal deaths in the post‐donation cohort studied by Garg and similar death rates between groups in the studies by Reisaeter and Yoo (2018).^21^, ^24^, ^30^ In contrast, Ibrahim reported an increase in fetal loss (OR: 1.83, 95% CI: 1.37–2.46) and premature deliveries (<36 weeks) (OR: 1.90, 95% CI: 1.03–3.48).^11^ However, fetal loss was reported as a composite of fetal death, miscarriage, and abortion. No statistical analysis is offered to compare fetal death specifically, though rates were lower in post‐donation pregnancies (2.1% vs. 4.9%).

### 3.4 The available guidance: what is recommend?

Table 3 lists the eight clinical practice guidelines,^31^, ^32^, ^33^, ^34^, ^35^, ^36^, ^37^, ^38^ three consensus statements,^39^, ^40^, ^41^ and four expert‐opinion papers,^42^, ^43^, ^44^, ^45^ all published between 2010 and 2020.

**TABLE 3:** Guidelines, consensus statements, and expert opinions written since 2010 and containing guidance on pregnancy risk in living kidney donors

Year of publication	Country/region	Funding body	Studies available at the time	Pre‐donation			Post‐donation	Recommendations
				Inquire about prior pregnancy complications	Inform of increased risk of post‐donation pregnancy complications	Seek alternative donors if the potential donor may still wish to have children	Specify post‐donation pregnancy follow‐up	
National/International Clinical Practice Guidelines								
Frutos and Cabello; Spanish Society of Nephrology (SEN) and Spanish National Transplant Organization (ONT) recommendations for living‐donor kidney transplantation^31^								
2010	Spain	SEN‐ONT	1,2,6–8	Yes	Informed consent form should state: “If you want to become pregnant after donation, you must inform your gynecologist as you are more likely to suffer from high blood pressure or diabetes during pregnancy.”		Yes. “Blood pressure, weight gain, and proteinuria must be closely monitored.”	“No additional risks have been described during the pregnancy of patients having donated a kidney… However, blood pressure, weight gain, and proteinuria must be closely monitored. In general, this is no different from what is normally recommended to any other pregnant woman.”
Richardson et al.: Canadian Kidney Paired Donation Protocol for Participating Donors 2014^32^								
2014	Canada	Canadian Blood Services' Living Donation Advisory Committee	1,2,6–8	Yes “[a] detailed description of the obstetrical history”.		Yes. [Exclude those] “with a history of toxemia in pregnancy and whose family is incomplete”.		Exclude from donation: “Premenopausal potential donor with a history of toxemia in pregnancy <10 years ago. [Those with] a history of toxemia in pregnancy in recurrent pregnancies. Premenopausal potential donor with a history of toxemia in pregnancy and whose family is incomplete”.
European renal best practice (ERBP); ERBP guideline on kidney donor and recipient evaluation and perioperative care 2015^33^								
2015	Europe	ERA‐EDTA	1–3,6–8	No	Yes	No	No	“There is no evidence for increased problems to conceive for women post/donation…. there is no evidence that nephrectomy results in serious adverse events during pregnancy. We recommend informing women of childbearing age that as they are selected from a very healthy subpopulation, donation increases their individual risk from below that of the general population, to that of the general population (1B)”
European Committee (Partial Agreement) on Organ Transplantation (CD‐P‐TO); Guide to the quality and safety of organs for transplantation^34^								
2018	Europe	Council of Europe / European directorate for the quality of medicines and healthcare	1–4, 6–10	No	Yes		No	Several studies have found that kidney donation is associated with increased incidence of hypertension as well as proteinuria. Females who have donated a kidney are at increased risk of pre‐eclampsia in subsequent pregnancies.
Lentine et al.; Summary of Kidney Disease: Improving Global Outcomes (KDIGO) Clinical Practice Guideline on the Evaluation and Care of Living Kidney Donors^35^								
2017		KDIGO	1–3,6–8, 10	Yes, “enquire about prior hypertensive disorders of pregnancy (e.g., gestational hypertension, pre‐eclampsia, or eclampsia).”	Yes, “We suggest that women with childbearing potential be counseled about the effects donation may have on future pregnancies, including the possibility of a greater likelihood of being diagnosed with gestational hypertension or preeclampsia.”	“Women should not be excluded from donation solely on the basis of a desire to have children after donation.”	“We advocate that recommended post‐donation pregnancy care be available to all women worldwide, including any treatments needed for pregnancy complications.”	“Women with a history of a prior hypertensive disorder of pregnancy (which includes pre‐eclampsia) may be acceptable for donation if the long‐term post‐donation risks are acceptable. A decision to proceed with donation in the year after childbirth should consider the psychological and health needs of mother and child.”
Andrews et al.; BTS/RA Living Donor Kidney Transplantation Guidelines 2018^37^								
2018	UK	British Transplantat‐ion society and Renal Kidney Association	1–4,6–10	No	Yes. “It is recommended that women are informed of a potential greater risk of pregnancy induced hypertension following kidney donation which may require specialist antenatal care, but it does not appear to lead to adverse outcomes for either mother or offspring”	No	“Close monitoring of blood pressure, creatinine, and fetal well‐being are advisable in kidneys donors during pregnancy.” “Kidney donors may be offered Aspirin 75 mg daily for pre‐eclampsia prophylaxis.” “Report births post‐donation to the Living Donor Registry as ”a significant medical event“ at each annual review”	“Women must be informed of a greater risk of pregnancy‐induced hypertension following kidney donation.” “There is no evidence to support the benefits of right or left nephrectomy to prevent pregnancy‐induced hydronephrosis.”
Wiles et al., UK Renal association clinical practice guideline; pregnancy and renal disease^38^								
2019	UK	UK Renal Association	1–12	NA	NA	NA	NA	We suggest kidney donors are offered low dose aspirin (75 mg‐150 mg) to reduce the risk of pre‐eclampsia (2D).
Organ Procurement and Transplantation Network (OPTN)^36^								
2020 (updated)			1–12	Yes, “enquire about gestational diabetes.”	“Disclose to all female kidney donors: Risks of pre‐eclampsia or gestational hypertension are increased in pregnancies after donation.”			“Risks of pre‐eclampsia or gestational hypertension are increased in pregnancies after donation.”
Consensus statements								
Maggiore et al.; Long‐term risks of kidney living donation: review and position paper by the ERA‐EDTA Descartes working group^39^								
2016	Europe	ERA‐EDTA Descartes	1–3,6–8		Yes			“Because the available studies point toward a somewhat higher risk of gestational hypertension and pre‐eclampsia, it is important to inform potential donors on this risk. It is, however, also important to note that such complications can be treated. In fact, overall outcome of pregnancies post‐donation in those studies was good.”
Lam et al.; Canadian Society of Transplantation and Canadian Society of Nephrology Commentary on the 2017 KDIGO Clinical Practice Guideline on the Evaluation and Care of Living Kidney Donors^40^								
2020	Canada	Canadian Society of Transplantation and Canadian Society of Nephrology	1–12	Yes. ‘For female donor candidates with a history of a hypertensive disorder of pregnancy, a detailed description should be obtained’			“Female donors should be counseled to maintain general good health, ensure adequate follow‐up, and receive proper pre‐pregnancy counseling and prenatal care.”	Donor candidates with a mild hypertensive disorder during pregnancy, or a single event that occurred more than 10 years ago who have normal eGFR, normal blood pressure, and no microalbuminuria and who have completed their families …can be considered for living kidney donation. Female donor candidates with recurrent episodes of preeclampsia/eclampsia during subsequent pregnancies … should be excluded from donation.
Mandelbrot et al.; KDOQI US Commentary on the 2017 KDIGO Clinical Practice Guideline on the Evaluation and Care of Living Kidney Donors^42^								
2020	US	National Kidney Foundation‐Kidney Disease Outcomes Quality Initiative (NKF‐NDOQI)	1–12	Yes “A careful pregnancy history that includes the presence, severity, and onset of preeclampsia is ..necessary in the evaluation of all female donors.”	Yes “Clinicians counseling these women can state that overall the rates of either gestational hypertension or preeclampsia increase from ~5% to 11%.”		“Individualized risk stratification is required and will influence appropriate pregnancy surveillance and preventative strategies. The need to share this portion of medical history with other members of the donor's health care team, especially obstetricians”	
Expert opinions								
Ahmadi et al. Shifting paradigms in eligibility criteria for live kidney donation: a systematic review^42^								
2015	the Netherlands		1–3,6–8					“On the basis of the literature that is available on this topic, there is no evidence to conclude women of childbearing age should be declined as potential kidney donors. However, one must bear in mind that comparison with the general population may be prone to confounding because live kidney donors are generally considered to be in better health. Most importantly the effects of donation on maternal and fetal outcomes should be part of the routine discussion about the risks of donation during the informed consent procedure. Quality of evidence available based on the GRADE tool was reported as poor. Level 4, grade of recommendation: C”
Sontrop & Garg; Considerations for LKD among women of child‐bearing age^43^								
2016	Canada	Expert opinion	1–3,6–8					“Potential donors with reproductive potential should be counseled on the possibility of a greater likelihood of GH or preeclampsia if they choose to donate a kidney but knowing that the probability of the most serious outcomes (stillbirth, neonatal death, maternal death) is extremely low.”
Hladunewich, Melamad and Bramham; Pregnancy across the spectrum of CKD^44^								
2016			1–3,6–8					“Female donors should be reassured.”
Lentine and Segev; Understanding and communicating medical risks for living kidney donors: A matter of perspective^45^								
2016	US		1–3,6–8					“For women of childbearing potential considering donation, we agree with recommendations of a recent AST LDCOP consensus statement that counseling should include the possibility of increased risk of gestational hypertension and pre‐eclampsia after donation compared with experience in otherwise similar healthy women. A balanced presentation, however, should also note that in available studies, most women had uncomplicated pregnancies after kidney donation.”

Four guidelines and two consensus statements recommended enquiring into previous pregnancy complications during the assessment of a potential LKD.^31^, ^32^, ^35^, ^40^, ^41^ Six guidelines recommended informing the potential LKD of the increased risk of post‐donation pregnancy complications.^31^, ^33^, ^34^, ^35^, ^36^, ^37^ One consensus statement suggested that alternative donors should be sought for women who had not completed their family and had a history of pre‐eclampsia.^40^ The 2017 KDIGO guidelines advised against excluding women solely on their desire to conceive post‐donation and suggested that women with a history of pre‐eclampsia may be suitable donor candidates.^35^ Three guidelines^31^, ^35^, ^37^ and two consensus statements^40^, ^41^ suggested increased or close follow‐up of pregnant LKD. The UK Renal Association guideline suggested all LKD were offered low dose aspirin to reduce the risk of pre‐eclampsia.^38^

Some guidelines gave conflicting information, for example, one guideline stated that “*no additional risks have been described during the pregnancy of patients having donated a kidney*’. However, they then specified that the consent form should state: ‘*If you want to become pregnant after donation, you must inform your gynecologist as you are more likely to suffer from high blood pressure or diabetes during pregnancy.*”^31^

The consensus from the expert opinions was that women should be counseled on the increased likelihood of gestational hypertension or pre‐eclampsia (Table 2).^39^, ^41^, ^42^, ^43^, ^44^, ^45^, ^46^

Mandelbrot et al. recommended quantifying this increased risk, advising clinicians state that “*overall the rates of gestational hypertension or pre‐eclampsia increase from ~5% to 11%.*”^41^ However, they continued by recommending that pregnancy care is tailored according to a woman's individualized risk of complications, taking into consideration the impact of race, BMI, and age on their risk of pre‐eclampsia.^41^ Lentine and Segev point out that potential LKD must also be informed that most women had uncomplicated pregnancies after kidney donation.^45^

## 4 DISCUSSION

We performed a systematic review to answer the question “*Are LKD at an increased risk of pregnancy‐induced complications following a donor nephrectomy, compared to the risks of pregnancy‐induced complications in healthy women who have not undergone a donor nephrectom*y*?*” We found nine relevant studies, the consensus from which was that the maternal risks associated with pregnancy in LKD increased from the pre‐donation risk. In relative terms, donation took LKDs from a risk level below that of the general population prior to kidney‐donation, to a risk level comparable to that of the general population post‐kidney donation. Risk of adverse fetal and neonatal outcomes was not different between post‐donation and pre‐donation pregnancies.

The main limitations of these studies were a low number of post‐donation pregnancies and event rates, the choice of relevant outcome measures, limited generalizability of the study groups, and the lack of suitable comparators. In the largest study using a before‐and‐after design, there were six cases of pre‐eclampsia and six of gestational hypertension in 173 post‐donation pregnancies.^22^ The largest matched‐cohort study by Garg reported on 131 post‐donation pregnancies, of which 15 were complicated by pre‐eclampsia and hypertension and eight babies were born before 37 weeks gestation.^24^

Wiles et al., demonstrated that hypertension (rather than eGFR) is the strongest predictor of pre‐term (<34 weeks) delivery (OR: 16.5, 2.74–∞) in women with CKD.^47^ Furthermore, they reported an increased likelihood of low birthweight infants born to women with proteinuric CKD.^47^ These pre‐pregnancy parameters were not reported in any of the studies. While, in the absence of proteinuria, LKDs are not considered to have CKD, screening for proteinuria and/or hypertension should form part of the pre‐pregnancy work up to better inform women of their risk of adverse fetal outcomes. This would also aid in identifying women who are likely to require increased surveillance in pregnancy for the development of superimposed pre‐eclampsia, given the challenge in diagnosing this condition in women with pre‐existing hypertension, proteinuria, and a single kidney. Overall, however, the equally low rates of fetal complications in post‐donation and pre‐donation or general population pregnancies can be used to provide relative reassurance to women undergoing LKD that their likelihood of placental disease affecting the fetus is unchanged by donation.

The generalizability of the results of these studies is limited to white women, as up to 98% were of this ethnicity (Table 1). Information on post‐donation risk of hypertensive complications of pregnancy in ethnic minority groups (EMG) cannot be inferred from the studies to date. Women from EMG are known to have a higher risk of pre‐eclampsia.^48^ Moreover, a recent review suggests that African American women with pre‐eclampsia experience more severe hypertension and increased mortality as compared to women of other ethnicities.^49^ As such, more studies documenting the pregnancy outcomes of non‐white LKDs are required to better inform post‐donation risk in this group.

The lack of a suitable comparator has been a particular problem when analyzing long‐term post‐donation risk. Until 2009, the studies by Buszta, Jones, and Wrenshall were the body of evidence with which guidelines on pregnancy risk in LKD were issued.^50^, ^51^ These studies compared the pregnancy outcomes of LKD to known outcomes in the unscreened general population. LKD are not a random subset of the population, rather an extensively screened group who are deemed to be at low risk of end‐stage kidney disease (ESKD). Given the overlap between risk factors for hypertensive complications of pregnancy, and those for ESKD (e.g., BMI >35 kg/m^2^, CKD, diabetes mellitus), comparison of LKDs to the general population is unlikely to yield accurate attributable risk.^35^, ^52^, ^53^, ^54^

To solve the conundrum of the adequate control group three studies included the pregnancy outcomes of the LKD both pre‐ and post‐donation. Although the before‐and‐after design is useful for assessing short‐term impacts, "threat to internal validity" may occur when assessing long‐term impacts. Over a longer period, one is more likely to develop a confounding condition which may obscure the effects of an intervention, for example, an increased BMI post‐donation. While the before/after design may appropriate risk more accurately than comparison to the general population, a more accurate comparator would be potential LKDs considered suitable for donation who did not proceed to donate. Accurate coding of these individuals in healthcare databases would allow for this group to be identified and their outcomes investigated.

The final limitation of all the studies reviewed was the absence of information on the LKD who did not have a post‐donation pregnancy and the reasons why. Therefore, potential medical or psychosocial consequences of donation that may have precluded pregnancy in LKDs remain unknown.

As a result of the small sample sizes and inconsistent comparator groups, we were unable to perform a meta‐analysis to provide a single estimate of effect. Our search for studies was comprehensive, albeit limited to English language studies. Furthermore, we do not strongly suspect publication bias as both negative and positive studies have been published.

Our second aim was to identify guidelines, consensus statements, and expert opinions which included the issue of pregnancy in LKD. While the guidelines offered albeit limited guidance on the acceptance of women of childbearing age as potential LKD, they were broadly consistent in stating that overall pregnancy after kidney‐donation was of a risk similar to that of the general population and that women should be informed of this risk. They varied in their guidance regarding enquiring into prior pregnancy‐induced complications, such as gestational diabetes and post‐donation pregnancy care.^34^ The 2017 KDIGO guidelines state that, “*women should not be excluded from donation solely on…a desire to have children after donation.*”^35^

More recent guidelines tended to be more comprehensive in their guidance, increasingly recognizing the importance of individualizing care. For example, as opposed to the blanket exclusion of women who have yet to complete their families, KDIGO and Canadian expert commentaries suggest that history of previous hypertensive disorders of pregnancy should be used to inform the prediction of individual post‐donation pregnancy risk and as such guide decisions as to potential LKD acceptability.^35^, ^40^ Similarly, expert consensus from the US published in 2020 suggests that care in post‐donation pregnancies should be tailored to individual risk.^41^ This is particularly important given the increasing rates of obesity and older‐age pregnancy, both factors recognized to increase risk of pregnancy complications.^42^

### 4.1 Communicating risks

Lentine and Segev propose that informed consent must be based on informing potential LKD of their baseline (pre‐donation) risk, the risk attributable to donation and their subsequent absolute (post‐donation) risk of adverse pregnancy outcomes.^45^

Baseline risk comprises demographic (e.g., age, ethnicity) and clinical (e.g., BMI, smoking status) characteristics, which in combination with the risk attributable to donation, can be used to calculate an absolute risk score for hypertensive complications of pregnancy. Established perinatal registries could be used to calculate the baseline risk of hypertensive disorders of pregnancy. To be able to combine both the baseline risk and the risk attributable to donation to create the absolute risk score, linkage of perinatal registry data to a robust large dataset, such as a prospective LKD registry is required. In an ideal scenario, the absolute risk of hypertensive pregnancy complications for the potential LKD would be derived by inputting baseline characteristics into a predicted risk calculator, such as www.transplantmodels.com.

Additionally, potential LKDs should be informed of the long‐term maternal consequences of pregnancy‐induced hypertensive complications. In the general population, pre‐eclampsia is associated with a greater than two‐fold risk of death from cardiovascular disease, a 5‐ to 9‐fold increased risk of ESKD and an increased risk of diabetes.^55^, ^56^, ^57^, ^58^ A recent study found that women with a pre‐donation pregnancy complicated by gestational hypertension had an increased risk of developing post‐donation hypertension, as compared to LKDs without pre‐donation gestational hypertension.^59^ Population‐based prospective LKD registries could be used to examine the association between post‐donation hypertensive disorders of pregnancy and risk of long‐term maternal cardiovascular and kidney disease to better inform potential donors.

Given the uncertainties surrounding post‐donation hypertensive complications in pregnancy, the communication of risk to potential LKDs remains challenging. In a recent survey of 392 transplant professionals from 30 countries, there was marked variability in the frequency with which adverse pregnancy outcomes were discussed. Fifty‐six percent of respondents always discussed pre‐eclampsia, 12% often did, 12% rarely did and 6% never discussed it. Only 20% of respondents were able to accurately answer questions on absolute versus relative risk for rare outcomes.^60^ While this survey may be subject to the inherent biases of self‐reports, it suggests there is a need to improve and standardize the communication of risk to potential LKDs. Box 1 lists our guidance on this topic, Box 2 lists our recommendations for future studies, and Box 3, provides a guide for practitioners counseling potential LKDs.

## 5 CONCLUSION

The increased risk of hypertensive disorders in pregnancy post‐donation is evident in four retrospective studies, although all were limited to some extent by sample size and the lack of well‐matched controls. Though the relative risk of pregnancy‐related complications in LKD increases relative to the risk in the non‐donor, the absolute risk remains very low. The risk of long‐term complications from hypertensive disorders in pregnancy is minimal.^57^, ^61^ Perhaps, the combination of these two factors is the reason why LKD guidelines vary in the degree of attention paid to this issue. The LKD of the future is likely to differ from the LKD of yesteryear. As such more focus should be placed on better identifying and individualizing risk for LKD in the face of both older age and higher BMI at kidney‐donation and subsequent pregnancy, and in non‐white LKD. Only by doing so will consent to donation be truly informed.

For now, one should keep in mind that a potential LKD's personalized risk remains unknown, however, the consensus is that a LKD could be reassured that the absolute risk of post‐donation pregnancy complications to mother and baby remains low.

BOX 1

1. Post LKD pregnancy risk needs to be personalized in light of the changing profile of the LKD who is increasingly older and more often overweight at the time of donation.
2. The additional post‐donation risk of pregnancy complications in LKD should always be seen in light of the national or regional baseline risk of adverse pregnancy outcomes.
3. The absolute risk of hypertensive‐pregnancy complications in LKD should be available in a risk‐stratified manner, for example, by age, BMI, and ethnicity, however, further studies powered to detect differences between these population sub‐sets are required to provide this information.
4. Informing potential LKD of their overall level of risk, taking into consideration the risks outlined in 1–3, should form part of the LKD consent process.
5. There should be uniformity in enquiring about previous pregnancy‐associated complications, that is gestational diabetes and hypertension, and neonatal complications while consenting for LKD.
6. Clearer guidance on LKD pregnancy follow‐up is needed. LKD should be secured follow‐up according to general international guidelines for pregnancies. Furthermore, they should be risk evaluated and referred to specialist obstetric care if deemed to be at increased risk.
7. It would be best to utilize more recent guidelines as those published prior to 2015 require revision.

BOX 2

1. Living kidney donor registries should collect the baseline characteristics required to calculate attributable risk of living kidney donation to hypertensive disorders of pregnancy, that is ethnicity, previous hypertensive disorders of pregnancy, and body mass index.
2. The baseline risk of hypertensive disorders of pregnancy should be determined using data held in perinatal registries.
3. Risk prediction models for hypertensive complications of pregnancy in LKD should be developed by linking LKD and perinatal registry data. One should accept that these registries, in the short term, may lack some variables required to individualize risk, that is, ethnicity.
4. LKD registries should prospectively collect data on suitable LKDs that did not proceed to donation. This will improve the quality of control groups required to understand long‐term LKD outcomes.
5. Qualitative research to understand prospective LKDs' concerns surrounding post‐donation pregnancy is needed.
6. The long‐term health consequences of hypertensive pregnancies in women post‐kidney donation should be established.

BOX 3 **A summary for practitioners counseling women on pregnancy post‐living kidney donation**

To date, 16 studies, published over a 35‐year period with follow‐up of 1399 post‐donation pregnancies have been performed. As all these studies are slightly different, we cannot say with precision what the average risk or risk to the individual woman and their baby is.

The pregnancy‐associated complications that have been most studied are hypertension in pregnancy and pre‐eclampsia. On average, the occurrence of hypertension in pregnancy changed from 1%–9% pre‐donation or in non‐donors to 4%–12% post‐donation. Pre‐eclampsia changed from 1%–3% pre‐donation or in non‐donors to 4%–10% post‐donation.

The risks to babies that have been reported are mainly limited to premature delivery, low birth weight, and transfer to the intensive care unit or death. Overall, there was no difference in the risk to babies born to kidney donors compared to the general population. Screening for pre‐pregnancy hypertension and/or proteinuria in post‐LKD women planning pregnancy can be used to individualize risk of adverse fetal outcomes.

## DISCLOSURE

The authors of this manuscript have conflicts of interest to disclose as described by the *American Journal of Transplantation*. Dr Maria Pippias and Dr Laura Skinner are currently funded by the National Institute for Health Research (NIHR). The review was not registered on Prospero. The review protocol can be supplied on request. An earlier version of this article has been published as part of a doctoral thesis and is currently under embargo.^62^

## Supporting information


Figure S1
Table S1

Appendix S1

Appendix S2

## Data Availability

Data sharing is not applicable to this article as no new data were created or analysed in this study.
