# Supplementary material for: Pregnancy after living kidney donation, a systematic review of the available evidence, and a review of the current guidance
Source: Am J Transplant. 2022 Aug 3;22(10):2360–80. doi: 10.1111/ajt.17122 (PMC9804926; doi:10.1111/ajt.17122)
Supplement: Supplementary file 1 — Figure S1 Table S1 [file AJT-22-2360-s002.docx]

Supplementary Figure 1. EMBASE search strategy as applied on 18^th^ December 2020

Search for: from 16 [limit 15 to (human and English language)] keep 1-52

Results: 52

Database: Embase Classic+Embase <1947 to 2020 December 18>

Search Strategy:

--------------------------------------------------------------------------------

1 maternal.mp. (370087)

2 fetal.mp. (313721)

3 pregnan*.mp. (1019204)

4 nephrect*.mp. (73083)

5 kidney.mp. or exp kidney/ (1314511)

6 renal.mp. (861945)

7 nephrol*.mp. (84640)

8 postdona*.mp. (372)

9 exp donor/ or donor.mp. (368981)

10 donation.mp. (41677)

11 exp donor/ (189148)

12 1 or 2 or 3 (1305454)

13 5 or 6 or 7 (1515406)

14 8 or 9 or 10 or 11 (379330)

15 4 and 12 and 13 and 14 (68)

16 limit 15 to (human and English language) (52)

17 from 16 keep 1-52 (52)

Supplementary Table 1 Pre-specified maternal and foetal outcomes extracted from the included studies

| **Participant variables** | **Maternal outcomes** | **Foetal outcomes** |
| --- | --- | --- |
| Median age at donation (years) | Proteinuria* | Pre-term birth (gestation <37/40 weeks) |
| Median age at pregnancy of interest (years) | Gestational hypertension* | Birth weight <2500 g |
| Median time from donation to pregnancy (years) | Pre-eclampsia* | Still birth |
| Body mass index (kg/m^2^) | Eclampsia* | Death <28 days after birth |
| Estimated glomerular filtration rate (ml/min/1.73m^2^) | Pre-eclampsia and gestational hypertension combined* | Other: as specified in study |
| Ethnicity (% white) | Gestational diabetes* |  |
|  | Death |  |
|  | Other: as specified in study |  |
| *as defined by the study in question.  Unclear information was not included. No assumptions were made regarding missing data. | | |
