# Supplementary material for: Pregnancy after living kidney donation, a systematic review of the available evidence, and a review of the current guidance
Source: Am J Transplant. 2022 Aug 3;22(10):2360–80. doi: 10.1111/ajt.17122 (PMC9804926; doi:10.1111/ajt.17122)
Supplement: Supplementary file 2 — Appendix S1 [file AJT-22-2360-s001.pdf]

Data extraction form: Pregnancy after living kidney donation, a systematic review and meta-analysis of the available evidence and a review of the current guidance.

Question to be addressed: *Are living kidney donors at an increased risk of pregnancy induced complications following a donor nephrectomy, compared to the risks of pregnancy induced complications in healthy women who have not undergone a donor nephrectomy?*

This data extraction form has been adapted from the Cochrane Consumers and Communication Group Data extraction template for included studies[1].

Please highlight any missing information as unclear or not described, to make it clear that the information was not included in the description of the study.

The inclusion criteria for the systematic review (and therefore data extraction) are as follows:

1. Population of interest: Living kidney donors with a post-donation pregnancy
2. Outcomes of interest: Pregnancy induced complications including but not limited too; gestational hypertension, gestational diabetes, pre-eclampsia, eclampsia, proteinuria
3. No limit on the follow-up time

If we proceed to a meta-analysis, then this will be performed on the studies which also include a comparator group. The comparator group(s) are living kidney donors with a post-donation pregnancy or healthy controls.

1. General information

1.	Date of form completed	
2.	Name of person completing form	
3.	Name of study (First_author_year)	
4.	Publication date (year)	
5.	Country in which the study was conducted	

2. Methods: Population and setting

		Descriptions as stated in the report/paper	Location in text
6.	Aim of the study		
7.	Study design (e.g., cross-sectional study, cohort study, before and after study)		
8.	Population description (from which study participants are drawn)		
9.	Number of arms or groups (including control groups); briefly describe each group		
10.	Geographic location i.e., country, rural, urban etc.		
11.	Number of transplant centres included in study		

12.	Data source(s)		
13.	Sampling technique (e.g., random or whole LKD population)		
14.	Period of kidney donation		
15.	Period of pregnancies		
16.	If survey: % of responders		
Notes:			

3. Results: Participant characteristics

		Descriptions as stated in the report/paper				Location in text
		Group 1:	Group 2:	Group 3:	Group 4:	
17.	Description groups e.g., group 1= LKD pre-donation, group 2= LKD post-donation, group 3= heathy control etc. (add more columns if necessary)					
18.	Total number of participants per group (n)					
19.	Total number of pregnancies per group (n)					
20.	Median age at donation (yr)					
21.	Median age at pregnancy of interest (yr)					
22.	Median time from donation to pregnancy (yr)					
23.	BMI (kg/m^2)					
24.	eGFR ($ml/min/1.73m^2$)					
25.	Ethnicity (% white)					
Notes:						

4. Outcomes

	Descriptions as stated in the report/paper	Location in text

26.	How were outcomes reported?		
27.	Please highlight key findings i.e., RR of XX for group x vs y		
Notes: **Please highlight the primary outcome if this was stated			

5. Results: Maternal Outcomes

Maternal outcomes, N* (%) *if other please state		Descriptions as stated in the report/paper				Location in text
		Group 1:	Group 2:	Group 3:	Group 4:	
28.	Proteinuria					
29.	Gestational hypertension					
30.	Pre-eclampsia					
31.	Eclampsia					
32.	Pre-eclampsia and gestational hypertension combined					
33.	Gestational diabetes					
34.	Death					
35.	Other:					
Notes:						

6. Results: Foetal Outcomes

Foetal outcomes, N (%) *if other please state		Descriptions as stated in the report/paper				Location in text
		Group 1:	Group 2:	Group 3:	Group 4:	
36.	Pre-term birth (gestation <37/40)					
37.	Birth weight <2500 g					
38.	Still birth					
39.	Death <28 days after birth					
40.	Other:					
Notes:						

7. Limitation and mitigation strategy

		Descriptions as stated in the report/paper	Location in text
41.	Strength		
42.	Limitation		
43.	Strategies to overcome the limitation		
44.	Bias; please mention any potential biases you have identified in this study		
Notes:			

8. Conclusion and other information

		Descriptions as stated in the report/paper	Location in text
45.	Key conclusions of study authors		
Notes:			

1. Ryan R, Synnot A, Prictor M, Hill S. Cochrane Consumers and Communication Group Data extraction template for included studies. CCCG http://cccrg.cochrane.org/author-resources. La Trobe University, Melbourne. Published November 2016. Approved (S. Hill) November 29th 2016. Accessed 6th December 2018.
